# Literary Notes

**Published:** 1903-09

**Authors:** 


### LITERARY NOTES.

---

Messrs. M. J. Breitenbach Company, importers of Gude's peptomangan, has sent out a chart containing 60 cuts in colors, showing pathogenic organisms, reproduced in great detail from water colors made by a competent bacteriologist. The *Medical News*, commenting upon this useful chart says:

"No textbook and no one work on pathogenic bacteria contains such a number of excellent diagnostic illustrations, nor such beautiful examples of lithographic art, as these. The full set of 60 cuts has been prepared to send to any physician who writes for them to the firm of M. J. Breitenbach Co., New York".

---

The International Journal of Surgery Company, 100 William street, New York, announces in press a book entitled, Nose and throat work for the general practitioner, by G. L. Richards, M. D., laryngologist to the Fall River Union Hospital, Fall River, Mass. Illustrated, bound in cloth, 375 pages; price $2.00.

---

The *Medical Mirror,* founded and edited by the late Dr. I. N. Love, is to be continued under the editorship of Dr. Ralcy Husted Bell. A corps of associated editors embracing some well-known names is announced on the cover.

---

The Buffalo State Hospital has issued its thirty-second annual report. The superintendent, Dr. Arthur W. Hurd, presents the affairs of the hospital in a clear and business-like manner, thereby giving further evidence of his capability and special adaptation to the work which he is called upon to perform. The total number of patients under treatment for the period covered by the report was 2,296, and when to this number is added force necessary to attend to the affairs of the institution, it will become apparent that it requires rare talent to properly administer the affairs of the hospital.

